# Cryoprecipitate utilisation, shortages and institutional policies in the United States: A survey of practices and policies

**DOI:** 10.1111/tme.70079

**Published:** 2026-04-07

**Authors:** Chinelo P. Onyenekwu, Mariam Ratiani, Jenna Wade, Angela Treml

**Affiliations:** ^1^ Department of Pathology Medical College of Wisconsin Milwaukee Wisconsin USA; ^2^ University of Wisconsin‐Madison Department of Pathology and Laboratory Medicine Madison Wisconsin USA; ^3^ Versiti Milwaukee Wisconsin USA

**Keywords:** ABO compatibility, blood component stewardship, cryoprecipitate, inventory management, shortages, transfusion practice

## Abstract

**Background:**

Since the COVID‐19 pandemic, demand for blood products has remained high, while donations have declined. Institutional policies guiding the use of these products may affect their availability, utilisation and waste. We surveyed blood banks (BBs) in the United States (U.S.) to assess policies and practices related to cryoprecipitate compatibility and to characterise experiences with administration, shortages and waste.

**Methods:**

An 11‐question online survey was distributed to U.S. BBs via Qualtrics. Responses were exported to Excel and analysed using descriptive statistics. Variables are reported as frequencies and percentages.

**Results:**

Of 241 BBs, 82 responded (34%). Thirty‐seven (45%) served community hospitals, 31 (38%) served academic hospitals and 55 (69%) served both adult and paediatric populations. Thirty (38%) BBs reported preferentially administering ABO type‐specific cryoprecipitate when available, although none reported restricting transfusion exclusively to type‐specific units under all circumstances. Eighteen (23%) limited the number of out‐of‐group units per patient within 24 h. Ten (13%) BBs reported shortages occurring at least 3–5 times per year. During shortages, 25 (61%) BBs proactively screened orders, and 14 (34%) used fibrinogen concentrate as an alternative. Annual wastage varied, with 12 (15%) BBs discarding more than 50 pooled units, often because unused units were thawed for massive transfusion protocols (MTPs). Strategies to reduce wastage included thawing units later in the MTP cycle, proactive order screening and supplementation with fibrinogen concentrate.

**Conclusion:**

Although a subset of BBs reported substantial shortages of cryoprecipitate, some institutions reported significant waste. Targeted strategies, such as limiting units administered, proactively reviewing orders, offering alternative products and modifying thawing policies for MTPs, may improve efficiency and reduce waste.

## 1 INTRODUCTION

Cryoprecipitated anti‐haemophilic factor (cryoprecipitate), a plasma‐derived product, is a crucial component of treatment for acquired hypofibrinogenaemia, trauma‐related coagulopathy, massive bleeding and certain obstetric emergencies. When frozen, it has a shelf life of 1 year, but once thawed, pooled units expire after 4 h in an open system or 6 h in a closed one. Each pre‐pooled unit of cryoprecipitate contains approximately 100 mL of plasma from 5 unpooled/single donor units of cryoprecipitate. Because of the residual donor plasma, cryoprecipitate may contain anti‐A and anti‐B isohaemagglutinins. A study by Hadjesfandiari and colleagues that investigated isohaemagglutinin titres in cryoprecipitate showed that these antibodies are not preferentially concentrated during cryoprecipitate production.^1^ They demonstrated that anti‐A and anti‐B titres in cryoprecipitate were comparable to those present in the corresponding plasma units, with median titres of approximately 1:32 and a modal titre of 1:16. In their analysis of the risk associated with transfusion of cryoprecipitate without consideration of ABO group, they suggested that the probability of transfusing a pooled adult dose of cryoprecipitate containing titres ≥1:100 is extremely low. Because the plasma volume transfused with cryoprecipitate is small relative to other plasma‐containing blood components, the risk of clinically significant haemolysis due to passive transfer of ABO antibodies is considered low in most circumstances.^1^, ^2^

Despite its longstanding clinical use, guidelines on ABO matching for cryoprecipitate are limited and often permissive. The AABB Standards^3^ and the Circular of Information^4^ state that ABO‐compatible cryoprecipitate is preferred when available, but it can be administered regardless of ABO type due to its low plasma volume and minimal risk of haemolysis. In 2022, discussions at a meeting of transfusion services experts in the United States suggested that practices regarding cryoprecipitate matching for ABO type may vary: some institutions routinely match ABO, others match only in specific cases and some use non–ABO‐matched units as standard. This variation has operational implications, especially as ongoing national supply pressures had caused many U.S. transfusion services (TS) to experience intermittent blood product shortages at the time.

The COVID‐19 pandemic revealed major vulnerabilities in healthcare systems, notably serious shortages of blood products such as cryoprecipitate, which emerged as a critically needed component.^5^ Blood supplies were severely affected by reduced donor participation due to pandemic‐related safety measures, limited donor sites resulting from an increase in remote working donors and reduced staffing at blood centres.^6^ Additionally, the long‐term decline in blood donations, especially among younger generations, has further exacerbated supply issues.^7^, ^8^ Since the pandemic ended, national organisations such as the American Red Cross (ARC) and America's Blood Centers (ABC) have issued urgent calls for action, with the ARC repeatedly noting a severe blood supply shortage.^9^, ^10^, ^11^ Despite awareness campaigns, blood donation rates have continued to decline.^12^ Uncertainties in supply and demand, along with the short shelf life of blood products, play a key role in the availability, usage and waste of blood components. In mid‐November 2025, data from ABC showed that 24 of 44 community blood centres across North America reporting their blood supply status had less than 3 days, with 16% (7/44) having a critically low level of less than 1 day. Blood centres require at least 3 days of blood supply to adequately meet their regular operational demands. Supplies with a 1‐day or less lead time are critically low and require urgent donations.^13^

In spite of these challenges, few studies have evaluated how U.S. hospitals handle ABO matching for cryoprecipitate, how often shortages or wastage occur, or what operational changes are made under supply constraints. Institutional policies requiring compatibility matching can affect the availability, demand and supply of cryoprecipitate. Enforcing ABO compatibility may result in supply shortages and increased waste. To fill this gap, we surveyed U.S. blood banks to understand their ABO matching policies, the frequency of shortages and wastage, and the adjustments made during limited‐supply periods. This aims to provide insights to improve utilisation and reduce cryoprecipitate waste.

## 2 MATERIALS AND METHODS

This multi‐institutional, questionnaire‐based survey of BBs in the U.S. was exempt from review by the Medical College of Wisconsin Institutional Review Board. No patients were involved in the study. A patient consent statement is not applicable or needed for this study. The survey was developed by a group of two transfusion medicine physicians and two pathology residents. Before distribution, the questionnaire was reviewed by two transfusion medicine physicians to assess clarity and content relevance, and it was modified based on their recommendations. The survey was deployed and collated via *Qualtrics* Software (Qualtrics, Provo, UT). It consisted of 11 questions: 9 structured questions with multiple‐choice responses and free‐text options and 2 open‐ended questions (Supplemental Material 1). The questions covered institutional demographics, cryoprecipitate use and administration practices, the frequency of supply/inventory shortages over the 2 years preceding the survey (February 2021 to February 2023) and annual cryoprecipitate wastage. For the purpose of this survey, a ‘unit’ of cryoprecipitate referred to a pooled or pre‐pooled unit of cryoprecipitate comprised of 5 unpooled (single donor) units of 15 to 20 mL. A shortage was defined as inventory below a 2‐day supply. Wastage was defined as cryoprecipitate units that expired prior to transfusion, including units that expired within the blood bank inventory and those that expired after issuance. Consistent use referred to institutions reporting that they restrict cryoprecipitate transfusion to ABO type‐specific units under all circumstances.

The estimated time to complete the survey was less than 15 min. The survey was deployed electronically through a network of blood bank medical directors in the US via an email list of BB directors and a directory of hospital transfusion services served by a regional blood centre in collaboration with the Blood Center's Hospital Relations Manager. It was distributed on February 7, 2023 and closed on February 21, 2023. Responses were voluntary. Responses were reviewed for completeness and internal consistency prior to analysis. All collected data were analysed in aggregate using *Qualtrics* Software (Qualtrics, Provo, UT) and *Microsoft Excel for Microsoft 365 Version 2303* (Microsoft Excel. Redmond, WA: Microsoft Corp; 2023).

## 3 RESULTS

The overall survey response rate was 34% (82/241). Nearly half (45%; 37/82) of respondents serve community hospitals, and 12% (10/82) serve a Level 1 trauma centre. Among BBs, 28% (22/78) serve hospitals with ≥500 beds, 69% (55/80) serve adult and paediatric patient populations and 79% (63/80) are in urban or suburban areas (Table 1).

**TABLE 1:** Demographics of surveyed blood banks.

	Responding blood banks, No. (%)
Type of institution served (multiple responses)	
Academic hospital	31 (38)
Community hospital	37 (45)
Level 1 trauma centre	10 (12)
Level 2 trauma centre	12 (15)
Level 3 trauma centre	7 (9)
Other (Veteran's Affairs, private facility)	4 (5)
Number of hospital beds (*n* = 78)	
<100	19 (24)
100–199	12 (15)
200–499	25 (32)
500–999	20 (26)
≥1000	2 (3)
Population served (*n* = 80)	
Adult only	24 (30)
Both	55 (69)
Paediatric only	1 (1)
Service setting (*n* = 80)	
Rural	17 (21)
Suburban	31 (39)
Urban	32 (40)

### 3.1 Cryoprecipitate policy and administration

Among BBs surveyed, although 30 (38%) reported preferentially administering ABO type‐specific cryoprecipitate when available, none reported restricting transfusion exclusively to type‐specific units under all circumstances. Eighteen (23%) limit the amount of cryoprecipitate administered to a patient within 24 h (Table 2). However, free‐text responses on the facility's cryoprecipitate policy reveal varied practices regarding the use of out‐of‐ABO‐group cryoprecipitate. Practices vary by recipient age, volume requirements and institutional policies. For neonates and infants, ABO compatibility is consistently prioritised in 100% (8) of respondents who reported on a paediatric‐specific policy, with 6 (75%) centres requiring type‐specific cryoprecipitate or, if the blood type is unknown, AB‐type cryoprecipitate (5, 63%). For adults, 8 (57%) facilities allow any ABO type, while 3 (21%) provide only ABO‐compatible units. Specific thresholds were also noted; for instance, one institution limits the use of more than 40 group O units for non‐O recipients within a 24‐h period. ABO matching is waived unless a substantial volume of cryoprecipitate is required (3, 21%), such as over 8 units in a single facility, or when a patient is identified as a large user in another facility. Criteria for defining a ‘large user’ were not provided. One TS reported restricting their inventory to types A and O cryoprecipitate only. Notably, two facilities indicated that ABO compatibility was not required for cryoprecipitate use, and one of them noted that it reserves AB‐type cryoprecipitate but rarely uses it for its paediatric population.

**TABLE 2:** Distribution of cryoprecipitate policy types, annual use, shortage and wastage among responding blood banks.

	Responding blood banks, No. (%)
Type of policy	
ABO‐unmatched	35 (44)
Type‐specific always	0 (0)
Type‐specific preferred	30 (38)
Other^a^	15 (19)
Existing limits on ABO non‐identical (*n* = 80) cryoprecipitate issued to a patient in a 24‐h period	
Yes	18 (23)
No	62 (77)
Annual usage in pooled units (*n* = 78)	
<100	43 (55)
100–500	14 (18)
501–1000	3 (4)
1001–2500	10 (13)
2501–5000	4 (5)
5001–7500	1 (1)
7501–10 000	1 (1)
>10 000	2 (3)
Annual frequency of shortage (*n* = 80)	
Never	39 (49)
Rarely (1–2 times)	27 (33)
Sometimes (3–5 times)	10 (13)
Often (6–12 times)	3 (4)
Very often (>12 times)	1 (1)
Average annual wastage in pooled units (*n* = 78)	
≤5	37 (47)
6–10	10 (13)
11–15	4 (5)
16–20	6 (8)
21–25	0 (0)
26–30	1 (1)
31–40	3 (4)
41–50	5 (6)
≥51	12 (15)

^a^ Responses included group AB cryoprecipitate for neonates/paediatrics, type‐specific cryoprecipitate for paediatrics.

### 3.2 Cryoprecipitate shortage and strategies

Fourteen (18%) BBs experienced cryoprecipitate shortages at least 3 times per year in the 2 years preceding the survey, while 4 (5%) facilities experienced shortages at least 6 times per year. Meanwhile, 39 (49%) centres reported no shortages during that period (Table 2), with some attributing their lack of shortages to low cryoprecipitate usage. Additional information from respondents revealed a variety of practices and experiences. Blood banks facing shortages (41, 100%) described strategies for managing product administration (Figure 1), such as medical screening and approval of all cryoprecipitate orders or multiple orders for the same patient (25, 61%). One facility reported implementing an interdisciplinary discussion process involving medical directors, pharmacy and the safety committee; 29% reported having standing orders from a secondary supplier or sharing resources by collaborating with neighbouring hospitals.

**FIGURE 1:**
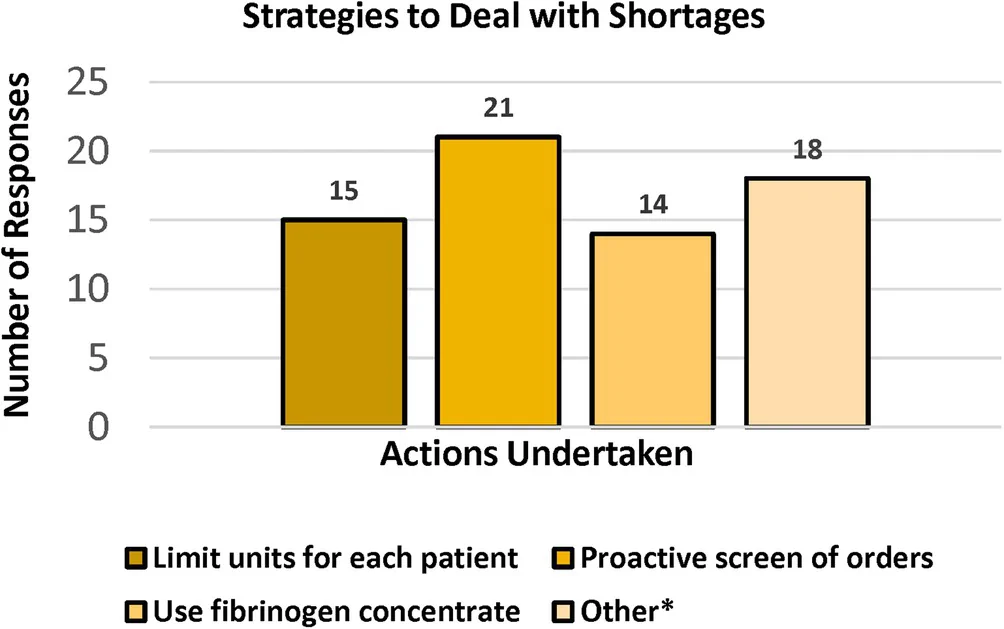
Strategies used to address cryoprecipitate shortages. *Include: Sourcing from neighbouring hospitals; placing a standing order with the secondary supplier; involving the pharmacy and safety committee and escalating to the medical director.

### 3.3 Cryoprecipitate wastage, strategies and additional information

Thirty‐seven (47%) respondents reported an average annual wastage of 5 or fewer pooled units, while 12 (15%) reported an annual wastage of 51 or more pooled units (Table 2). In providing additional information on the survey, 6 of 7 respondents (86%) who commented on wastage noted that waste occurred primarily because units thawed for massive transfusion protocols (MTP) were left unused by the ordering department. One BB reported that screening before thawing mitigates wastage. BBs also reported adjusting their policies to minimise waste; one BB reported changing to thawing cryoprecipitate only after the third MTP cycle had been dispatched, resulting in less waste, as the median number of MTP cycles at the facility was 2. Four of 19 (21%) respondents who provided additional information mentioned the use of pathogen‐reduced cryoprecipitate (also called INTERCEPT Fibrinogen Complex or IFC) and fibrinogen concentrate. For one respondent, approximately 30% of their cryoprecipitate usage was supplemented by IFC, which has a 5‐day shelf life, significantly reducing wastage. Additionally, three (19%) respondents stated that their facilities planned to switch to IFC and fibrinogen concentrate.

## 4 DISCUSSION

This national survey of U.S. TS highlights institutional practices related to cryoprecipitate compatibility policies, inventory management and wastage patterns across TS during the study period of February 2021 through February 2023. Several key observations emerged from the survey. First, although 38% of institutions reported a preference for administering ABO type‐specific cryoprecipitate, none reported restricting transfusion exclusively to type‐specific units under all circumstances. This reflects a pragmatic balance between compatibility considerations and inventory constraints. Second, reported experiences with cryoprecipitate shortages varied across institutions; while nearly half of respondents reported at least one shortage during the survey period, frequent shortages were uncommon, occurring in 18% of respondents. Third, substantial heterogeneity was observed in reported wastage patterns, suggesting differences in institutional inventory management strategies and clinical utilisation practices. Together, these findings highlight the variability in cryoprecipitate practices across institutions and emphasise opportunities to improve standardisation and optimise inventory management. The variability in cryoprecipitate practices observed in this survey is consistent with prior reports demonstrating heterogeneous utilisation across institutions. Multicentre data from the Biomedical Excellence for Safer Transfusion (BEST) Collaborative showed substantial differences in cryoprecipitate dosing and departmental utilisation across North American hospitals, suggesting that local practice patterns and product availability influence utilisation.^14^ Earlier audits have similarly reported marked variation in cryoprecipitate use and adherence to recommended indications. In a Canadian multicentre audit of 25 hospitals, cryoprecipitate utilisation ranged from 2 to 27 units per 100 red blood cell units transfused, and only 24% of transfusions were classified as appropriate according to published guidance.^15^ Operational studies suggest that institutional workflow and oversight may influence cryoprecipitate wastage; removal of prospective transfusion medicine approval has been associated with increased discard rates, whereas targeted education and process changes have been shown to substantially reduce wastage.^16^, ^17^ Wastage rates of 7.7%–16.7% have been reported in operative settings.^16^, ^18^ The findings of the present survey extend this literature by examining institutional policies related to ABO compatibility, cryoprecipitate shortages, rationing strategies and wastage, areas that have been less described in prior studies.

### 4.1 Impact of blood bank type and patient population on cryoprecipitate policies

The distribution of responding BBs is similar to the diversity of settings in which cryoprecipitate is used. Notably, the majority of surveyed BBs serve community hospitals, followed by academic hospitals. Institutional context may influence the strategies employed in cryoprecipitate inventory management. Academic medical centres and community hospitals often differ in transfusion volume, laboratory infrastructure and clinical service complexity. These factors may shape how transfusion services approach ABO compatibility policies and cryoprecipitate inventory management.

Most BBs in this study serve both adult and paediatric populations, which can complicate blood product administration. Paediatric patients often require more precise ABO matching and individualised dosing of clotting factors, mainly due to their smaller total plasma volumes and unique physiology and developmental haemostasis.^19^, ^20^, ^21^, ^22^ This often leads to a more cautious approach to cryoprecipitate use, adding further challenges to supply management during shortages.

### 4.2 Preferences for type‐specific cryoprecipitate and administration policies

Favouring issuing ABO type specific cryoprecipitate is resource‐intensive, may result in waste during periods of low demand, and can exacerbate supply shortages if demand increases or type‐specific cryoprecipitate supply becomes limited. This reported preference highlights the challenge for BBs to balance optimal patient treatment with resource management during crises.

Limiting out‐of‐group cryoprecipitate reflects a trend across health care to enforce stricter controls on blood product usage to ensure patient safety. Unlike red blood cell transfusions, where ABO incompatibility can be fatal, there is less emphasis on ABO matching for platelet and plasma transfusions.^23^ The primary concern has been avoiding haemolysis. Plasma containing blood components, including cryoprecipitate, contains anti‐A and anti‐B antibodies, which can occasionally trigger haemolytic transfusion reactions that may sometimes be severe or rarely fatal.^24^, ^25^, ^26^

While TS have policies on ABO‐incompatible plasma in blood products such as platelets and cryoprecipitate, no definitive method exists to mitigate the risks associated with anti‐A and anti‐B antibodies. This leaves a residual risk of haemolysis when large quantities of such products are administered. The wider impact of ABO‐minor mismatched plasma transfusions remains uncertain but concerning. Soluble A and B antigens present in some plasma units (notably group AB) can bind to recipients' circulating anti‐A/anti‐B antibodies and form circulating immune complexes, potentially causing pro‐inflammatory and pro‐thrombotic effects. Evidence links exposure to ABO‐compatible (non‐identical) plasma with increased rates of acute complications (such as acute respiratory distress syndrome, and sepsis),^27^ possible adverse effects,^28^, ^29^ and in large registry studies, a small but statistically significant rise in short‐term mortality, particularly with repeated plasma exposure.^30^ Notably, AB plasma, traditionally seen as a universal donor, contains both A and B soluble antigens. When transfused to recipients with anti‐A and/or anti‐B antibodies, especially type O individuals, clinically significant antibody–antigen interactions can occur. Shanwell et al. reported an adjusted relative risk of 1.15 (95% CI 1.02–1.29) for death after 5 or more units of ABO‐compatible but non‐identical plasma.^30^ For cryoprecipitate, the clinical significance of plasma volume is debated and may be particularly relevant in patients with smaller body weight and lower blood volumes, such as neonates and children. This could explain the observed preference for type‐specific cryoprecipitate administration in this survey population. Limiting out‐of‐group cryoprecipitate could lead to suboptimal care if ABO‐identical units are unavailable, especially during shortages. This presents ethical dilemmas for BBs. Of note, several observational studies and reviews suggest no clear association between ABO‐mismatched platelet and plasma transfusions and increased mortality.^31^, ^32^, ^33^

### 4.3 Blood bank strategies during cryoprecipitate shortages

Half of the responding BBs reported experiencing a cryoprecipitate shortage in the 2 years before the survey; however, most respondents indicated that shortages occurred infrequently (less than 3 times in a year), with 18% facing shortages at least 3–5 times per year. This pattern indicates that cryoprecipitate supply issues were ongoing towards the end of the pandemic period, rather than isolated incidents, highlighting the need for more robust, sustainable management strategies. In response to shortages, proactive screening of orders which involves close collaboration between clinical teams and BB staff guarantees that only necessary transfusions occur. Nonetheless, this strategy may also delay treatment for some patients, potentially resulting in less optimal care in urgent, high‐risk situations.

Limiting the number of units administered to each patient is another approach to conserving the inventory. While this can help to preserve resources, it may also result in the under‐treatment of patients, especially those with severe bleeding disorders who require higher doses of cryoprecipitate for optimal coagulation. Consequently, close monitoring is essential to prevent this strategy from compromising patient outcomes.

Among BBs, a notable number reported using fibrinogen concentrate as an alternative to cryoprecipitate, highlighting a trend towards other clotting factor products during shortages. Fibrinogen concentrate, which contains fibrinogen, a key clotting factor in the coagulation cascade, may be a viable substitute for cryoprecipitate in certain bleeding scenarios due to its quick reconstitution, viral inactivation and predictable dosing.^34^, ^35^ However, its use requires careful clinical judgement, as randomised trials and systematic reviews show limited high‐quality evidence of a mortality benefit, and risks such as thrombosis at higher fibrinogen targets.^36^, ^37^, ^38^ Importantly, fibrinogen concentrate lacks other coagulation factors, like factor VIII, and is thus not a direct replacement for anti‐haemophilic factor in patients with haemophilia A; guidelines still recommend specific factor VIII concentrates for these patients. Pathogen‐reduced cryoprecipitated fibrinogen complex (also known as INTERCEPT Fibrinogen Complex or IFC) is increasingly used during shortages as a supplement or alternative to traditional cryoprecipitate because it is United States Food and Drug Administration (FDA)‐approved for fibrinogen replacement and can be stored at room temperature for up to 5 days after thawing, allowing immediate use in bleeding emergencies.^39^, ^40^, ^41^ Lu et al. demonstrated significant variability in cryoprecipitate use across North American academic hospitals, with fivefold differences in doses per patient and inconsistent thresholds for fibrinogen replacement. They also noted that cryoprecipitate distribution in the U.S. continues to rise despite international shifts towards standardised fibrinogen concentrates and pathogen‐reduced products. The authors emphasise that practice is influenced more by cost, availability and clinician preference than by evidence.^14^ Data from an early implementation study on IFC (manufacturer report) report operational benefits such as reductions in cryoprecipitate waste from 16.3% to 5.0% in a paediatric centre.^41^ Reductions from 8.8% to 2.4% have also been reported across multiple sites, with significant improvements in turnaround times, and a 58% reduction after IFC adoption.^18^, ^39^ However, the practical deployment of IFC varies widely due to differences in acquisition costs, contracting practices and product availability, which can negatively impact the operational benefits. Clinical outcome data remain limited, primarily based on functional assays and initial observational studies rather than large prospective trials.^40^, ^42^ While IFC offers operational advantages, its broader use depends on local costs, supply stability and institutional clinical priorities.

### 4.4 Cryoprecipitate wastage: Causes and management

One of the most notable findings of this study is the wide variation in cryoprecipitate wastage among responding BBs. While nearly half of the participants reported wasting no more than five pre‐pooled cryoprecipitate units annually, a considerable proportion reported wasting over 50 units. This variation can be linked to factors such as differing inventory management practices, institution size and protocols for thawing and use. Higher wastage often occurred when thawed units were not used, especially during MTPs. In these situations, due to its lengthy thaw time and approximately 30‐min turnaround time, cryoprecipitate units are often thawed in anticipation of potential needs but are not ultimately used because of patient stabilisation or changing clinical conditions. Due to the short post‐thaw half‐life, BBs find it difficult to use most of the thawed units for MTPs. Delaying the thaw of cryoprecipitate until the third cooler may reduce wastage; however, this approach could delay treatment in traumatic haemorrhage cases requiring immediate massive transfusion and cryoprecipitate. Dunbar et al., in their survey of three Level 1 trauma centres, found that cryoprecipitate had the highest wastage rate across all three sites.^43^ While issuing more products than needed during MTPs is understandable to avoid under‐resuscitation and potential increases in patient mortality or morbidity, our survey suggests that delaying cryoprecipitate thawing until after issuing the second set of blood products may help reduce wastage. Some BBs also use IFC and fibrinogen concentrate as supplements or alternatives to cryoprecipitate during shortages, offering another way to reduce wastage. The variability in cryoprecipitate compatibility policies and inventory management practices observed in this survey and in a previous report^14^ occurred amid changing fibrinogen replacement strategies in transfusion medicine. In several regions, especially in Europe, fibrinogen concentrate has increasingly been used as an alternative to cryoprecipitate. However, cryoprecipitate remains the main fibrinogen replacement product in many countries, including the United States.^44^ As newer products like pathogen‐reduced cryoprecipitate and fibrinogen concentrate become more accessible, transfusion services may reconsider institutional policies on cryoprecipitate compatibility, inventory management and waste reduction. Therefore, understanding existing institutional practices provides an important baseline for assessing potential changes in fibrinogen replacement strategies.

This study has several limitations. The survey was conducted between February 2021 and February 2023, and TS practices and policies may have changed since then. In addition, the survey period overlapped with the COVID‐19 pandemic, during which blood supply disruptions and operational pressures may have influenced cryoprecipitate inventory management and utilisation practices. Consequently, the findings should be interpreted as reflecting practices during the survey period rather than the current practice at responding institutions. The respondents' geographic locations were not collected, preventing analysis of regional differences in policies, shortages or supply constraints. The survey relied on respondents' adherence to instructions not to share the survey link within their institutions. No other measures were implemented to prevent multiple submissions, so duplicate responses cannot be definitively excluded. Clinical outcomes associated with specific ABO matching approaches were not evaluated. Wastage data were reported as the number of pooled units wasted per annum and the percentage of facilities experiencing wastage, rather than as a proportion of total inventory or as the number of cryoprecipitate doses wasted, which limits cross‐institutional comparisons. Additionally, because the survey was designed primarily to characterise institutional policies and practices, shortages and wastage were not assessed against each institution's specific characteristics such as trauma designation, cryoprecipitate policies or procedures, preventing an evaluation of how local workflows and demographics may influence the reported outcomes.

Notwithstanding these limitations, this study has notable strengths. It presents one of the few snapshots of cryoprecipitate management across U.S. TS during both normal supply periods and times of sustained supply challenges. The survey adds to the existing literature by providing information on institutional ABO‐matching policies for cryoprecipitate. The practice‐level detail reported by respondents strengthens the relevance of the findings and stresses the need for harmonised national practices that reduce waste and improve resilience during shortages.

This study highlights the essential role that BB policies, particularly those related to compatibility testing and product administration, play in managing cryoprecipitate supply, use and waste during shortages. Although most BBs aim to enhance patient safety through type‐specific cryoprecipitate delivery and rationing, the remarkable variation in wastage rates indicates opportunities to improve inventory control and operational efficiency. The results also emphasise the importance of alternative clotting factor products, such as fibrinogen concentrate and IFC, in addressing supply shortages, though these alternatives present their own challenges. Though the study focuses on U.S. practice, the findings may be informative for international health systems in which cryoprecipitate remains in use or where fibrinogen replacement strategies are evolving, particularly with respect to how permissive guidance, supply constraints and local policy shape transfusion practice.

In summary, policies and practices for cryoprecipitate vary widely across U.S. institutions. Reported shortages and waste reflect variable practice patterns during supply chain issues. These findings highlight opportunities to standardise cryoprecipitate stewardship, improve operational workflows and establish evidence‐based national guidance to enhance clinical use and inventory management.

*Supporting Information*: Supplemental Material 1 includes the survey questionnaire, which consists of 11 questions used to collect data on institutional cryoprecipitate use, compatibility practices, inventory management, experiences with shortages and wastage.

## AUTHOR CONTRIBUTIONS

Conceptualisation and study design: Angela Treml and Jenna Wade. Development of survey instrument: Chinelo P. Onyenekwu, Mariam Ratiani, Jenna Wade and Angela Treml. Coordination of data collection: Mariam Ratiani. Data analysis and interpretation: Chinelo P. Onyenekwu, Mariam Ratiani, Jenna Wade and Angela Treml. Methodological oversight: Angela Treml and Jenna Wade. Original manuscript draft: Chinelo P. Onyenekwu. Manuscript review: Mariam Ratiani, Angela Treml and Jenna Wade.

## CONFLICT OF INTEREST STATEMENT

The authors have no competing interests.

## PATIENT CONSENT STATEMENT

Patient consent statement is not required for this study.

## Supporting information


**Data S1.** Supplemental Material.

## Data Availability

The data that support the findings of this study are available from the corresponding author upon reasonable request.
