# Supplementary material for: Cryoprecipitate utilisation, shortages and institutional policies in the United States: A survey of practices and policies
Source: Transfus Med. 2026 Apr 7;36(5):503–10. doi: 10.1111/tme.70079 (PMC13670394; doi:10.1111/tme.70079)
Supplement: Supplementary file 1 [file TME-36-503-s001.docx]

Supplemental Material 1.

**Cryoprecipitate Usage and Compatibility Practices Among Blood Banks in the United States**

1. What option best describes your blood bank? (Multiple responses may be applicable)
A) Blood bank serving an academic hospital/medical center
B) Blood bank serving a community hospital
C) Blood bank serving a private facility
D) Blood bank serving a level 1 trauma center
E) Blood bank serving a level 2 trauma center
F) Blood bank serving a level 3 trauma center
G) Other (please specify)__________________
2. How many beds does your blood bank serve? (Please list bed number for the largest facility if multiple facilities are served) ………………
3. What best describes your setting?
   a. Urban
   b. Suburban
   c. Rural
4. What population do you serve?
A) Adult only
B) Pediatric only
C) Adult and pediatric
5. What type of cryoprecipitate policy is practiced at your facility?
A) Cryoprecipitate never matched for blood type prior to administration
B) Type specific cryoprecipitate administered always
C) Type specific is preferred
D) Other (please specify)____________________
6. Do you limit the number of out-of-group cryoprecipitate units given within a 24-hour period?
   a. Yes
   b. No
7. What is your annual cryoprecipitate usage?
A) <100 units
B) 100-500 units
C) 501-1000 units
D) 1001-2500 units
E) 2501-5000 units
F) 5001-7500 units
G) 7501-10,000 units
H) >10,000 units
8. How frequently have you experienced cryoprecipitate shortage (less than 2-day supply in stock) in the past two years?
A) Never
B) Rarely (1-2 times per year)
C) Sometimes (3 -5 times per year)
D) Often (6 – 12 times per year)
E) Very often (>12 times per year)
9. If you have experienced a cryoprecipitate shortage (less than 2-day supply in stock) in the last two years, did you? (Select as many options as needed)
   a. Limit number of cryoprecipitate units given to a patient at a time
   b. Proactively screen orders
   c. Use fibrinogen concentrate in place of cryoprecipitate
   d. Other (please specify)
10. On average what is your yearly cryoprecipitate wastage?
    a. < 5units
    b. 6-10
    c. 11-15
    d. 16-20
    e. 21-25
    f. 26-30
    g. 30-40
    h. 40-50
    i. >50
11. Is there anything you would like to share about your cryoprecipitate usage or practice?

_____________________
